# Impact of a Mandatory Prescription Drug Monitoring Program on Prescription of Opioid Analgesics by Dentists

**DOI:** 10.1371/journal.pone.0135957

**Published:** 2015-08-14

**Authors:** Linda Rasubala, Lavanya Pernapati, Ximena Velasquez, James Burk, Yan-Fang Ren

**Affiliations:** Department of General Dentistry, University of Rochester Eastman Institute for Oral Health, 625 Elmwood Avenue, Rochester, New York, 14620, United States of America; University of Pittsburgh, UNITED STATES

## Abstract

Prescription Drug Monitoring Programs (PDMP) are statewide databases that collect data on prescription of controlled substances. New York State mandates prescribers to consult the PDMP registry before prescribing a controlled substance such as opioid analgesics. The effect of mandatory PDMP on opioid drug prescriptions by dentists is not known. This study investigates the impact of mandatory PDMP on frequency and quantity of opioid prescriptions by dentists in a dental urgent care center. Based on the sample size estimate, we collected patient records of a 3-month period before and two consecutive 3-month periods after the mandatory PDMP implementation and analyzed the data on number of visits, treatment types and drug prescriptions using Chi-square tests. For patients who were prescribed pain medications, 452 (30.6%), 190 (14.1%), and 140 (9.6%) received opioid analgesics in the three study periods respectively, signifying a statistically significant reduction in the number of opioid prescriptions after implementation of the mandatory PDMP (p<0.05). Total numbers of prescribed opioid pills in a 3-month period decreased from 5096 to 1120, signifying a 78% reduction in absolute quantity. Prescriptions for non-opioid analgesics acetaminophen increased during the same periods (p<0.05). We conclude that the mandatory PDMP significantly affected the prescription pattern for pain medications by dentists. Such change in prescription pattern represents a shift towards the evidence-based prescription practices for acute postoperative pain.

## Introduction

In the United States the amounts of prescription opioids have increased significantly in the past two decades, from 75.5 million in 1991 to 209.5 million in 2010 [1]. US health professionals dispensed enough opioid drugs for every American to take an equivalent of 5mg of hydrocodone 6 times daily for 1 month 2010 [2]. Unintended deaths by prescription opioid overdose have quadrupled in the same time period and become the leading cause of overdose ahead of heroin and cocaine [1, 3]. Prescription opioid drugs were involved in three quarters of the drug overdose deaths in the United States, mounting to 12 deaths annually per 100,000 population [2].

To curb the abuse of opioid drugs and reduce unintended opioid overdose deaths, 49 states to date have instituted prescription drug monitoring programs (PDMP), which are statewide electronic databases that collect designated data on substances dispensed in that state [4, 5]. A PDMP collects information on all Schedule II, III and IV controlled substances received by an individual in the past 6 months, including the name, dosage and quantity of the drugs, date for each prescription, and name and title of the prescriber. With this data, an individual who seeks opioid drugs from different providers may be easily identified and prevented from receiving multiple opioid prescriptions at the same time. Authorities in most states encourage healthcare professionals to voluntarily consult the PDMP database before prescribing controlled drugs. In August 2013, New York became one of the first states to mandate that prescribers consult the state's PDMP registry before prescribing a controlled substance. The New York State Internet System for Tracking Over-Prescribing, or iSTOP, creates a real-time system to check a patient’s recent prescription history to determine whether he or she exhibits drug-seeking activities [6]. The iSTOP program is especially helpful to healthcare professionals who are not familiar with the individual’s medical history, such as emergency room physicians and urgent care dentists, who will be able to check the real-time database and determine if a similar prescription already exists for an individual who is seeking opioid drugs.

It has been shown that the PDMP may help healthcare professionals identify drug-seekers and reduce inadvertent prescription of opioids to these patients [7, 8]. As the PDMP is a voluntary program in most states, its utilization by healthcare providers has been inconsistent over the past years [9, 10]. Though several states are now requiring mandatory consultation of the PDMP databases before prescribing controlled substances [4], its effect on reducing the number of prescriptions for opioid analgesics remains unknown.

Our dental urgent care center is dedicated to treating patients with acute dental pain, infection and dentoalveolar traumas in the Greater Rochester area in New York. Approximately 650 patients visit our dental urgent care center monthly. Our internal data showed that more than 70% of these patients had acute dental pain that required a tooth extraction or root canal therapy, and at least 25% of them received opioid analgesics prior to August 2013, when the mandatory PDMP was implemented. Due to the large number of prescriptions for opioid analgesics to a patient population that is transient in nature, the urgent dental care center is an ideal setting for assessing the effect of the mandatory PDMP on prescribing opioid analgesics. We hypothesized that the mandatory PDMP would significantly reduce the number of prescriptions for opioid analgesics by dentists. To test this hypothesis, we conducted a series of cross-sectional analyses of patient records in the urgent dental care center and compared the prescription patterns for pain medications before and after implementation of the mandatory PDMP.

## Materials and Methods

This study was approved by the Research Subject Review Board of the authors’ institution (approval number RSRB00051884). All patient information were anonymized and de-identified prior to the analysis.

### Data collection

This study was designed as a cross-sectional survey of patient records in a dental urgent care center before and after the mandatory PDMP implementation in New York State. As the deadline for implementing the mandatory PDMP was August 27, 2013, we surveyed a 3-month period after this date and compare the data with those of a 3-month period before this date. To avoid inconsistencies associated with the initial implementation of the program, we avoided the first 3-month period immediately following the August 27, 2013 deadline as there might be irregularities caused by system, enrollment or utilization errors of the online program. Records of all the patients who visited the urgent care center between December 1, 2013 and February 28, 2014 were analyzed and compared with those between the same time period the prior year (December 1, 2012 to February 28, 2013). To study the consistency of the effect of mandatory PDMP on drug prescriptions, records of all the patients who visited the urgent care center during the 3-month period immediately after February 2014 were also analyzed.

The following information was retrieved from the electronic records of every patient who visited the dental urgent care center during the three selected 3-momth periods (Pre-iSTOP—12/1/2012 to 2/28/2013, Post-iSTOP-1–12/1/2013 to 2/28/2014, and Post-iSTOP-2–3/1/2014 to 5/31/2014):

1. Total number of patient visits: Patients were either in the urgent care center for an initial visit (ADA code D0140, problem oriented exam), or for a follow-up or observation visit after a procedure performed during a previous visit (ADA code D0113, 0115, 0117 and D9430).
2. Procedures performed by ADA treatment codes: Only frequently performed procedures were included. Extractions were divided into simple extractions (D7410) or surgical extractions (D7210, and D7220-7240 for impacted teeth). Other procedures included therapeutic pulpotomy (D3220), incision and drainage of intra-oral abscesses (D7510), and palliative treatment for dental pain (D9110).
3. All medications prescribed, including drugs, dosages and quantities (number of pills): Medications included opioid and non-opioid analgesics, antibiotics, and antimicrobial rinses. As the primary aim of the present study was on the impact of the mandatory PDMP on prescription of opioid analgesics, we focused our analysis on Schedule II and III drugs that include hydrocodone, oxycodone and codeine in its formulations.

### Sample size estimate and data analysis

Our pilot data indicated that at least 25% of the patients who visited the dental urgent care center would receive Schedule II or III pain medications prior to the implementation of the mandatory PDMP in August 2013. Assuming that a 20% reduction (from 25% to 20%) in prescription of opioid analgesics signifies that the PDMP has produced a significant effect, we needed 1,464 subjects each before and after the implementation to achieve 90% power at an alpha level of 0.05. As we had on average 650 patient visits per month, we decided that patient data from a 3-month period would satisfy the sample size requirements. Records of all the patients who visited the dental urgent care center during the three study periods were included in the analyses. Frequencies of the procedures and prescriptions were compared among the three study periods using Chi-square contingency table tests. For patients who received analgesics, we compared the odds of receiving opioids with that of receiving non-opioids and used odds ratio (OR) and its 95% confidence interval (CI) to estimate the impact of mandatory PDMP on prescription of opioid analgesics. We found that the treatment procedure invasiveness varied in the three study periods as the frequency of surgical extraction was higher in the Pre-iSTOP period, adjustment was therefore made to account for the effect of treatment procedures, and adjusted OR was calculated for Post-iSTOP-1 and Post-iSTOP-2. Descriptive statistics was used to show the change in the number, frequency and quantity of opioid analgesic prescriptions before and after mandatory PDMP implementation. All statistics were performed using *OpenEpi* (Version 3.03) software for two-tailed tests, and a p-value of less than 0.05 was considered statistically significant.

## Results

A total of 1921, 2011 and 2272 patient visits were registered in the patient electronic records during the three 3-month periods, respectively. Most of these visits were classified as an “initial visit” for a specific dental problem. There were no statistically significant differences in the number of visits classified as a “follow-up” for a dental problem that was previously treated in the same clinic (5.8%, 4.3%, and 4.8% for the three study periods, respectively, p>0.05) (Table 1). The most frequently performed treatment procedures were dental extractions, where 1188, 984, and 1089 teeth were extracted in the three 3-month periods, respectively.

**Table 1:** Frequencies of visit types, treatment procedures and prescriptions for dental emergency patients before and after implementation of the mandatory PDMP.

		Pre-iSTOP	Post-iSTOP-1	Post-iSTOP-2	p-value	
	Total visits	1921	2011	2272		
**Exam**	Initial visit (D140)	1810	1924	2163		
	Follow up (D0113, 15,17, D9430)	111	87	109	0.1014	ns
**Treatment**	Simple extraction (D7140, D7250)	782	727	820	0.0879	ns
	Surgical extraction (D7210-40)	406	257	269	<0.0001	*
	Pulpotomy (D3220)	89	90	73	0.0576	ns
	Incision and Drainage (D7510)	15	10	11	0.4584	ns
	Palliative treatment for pain (D9110)	204	188	228	0.6320	ns
**Prescription**	Opioids analgesics	452	190	140	<0.0001	*
	Non-opioids analgesics	1023	1158	1317	0.2077	ns
	Antibiotics	665	738	854	0.1288	ns
	Chlorhexidine (0.12%)	192	199	248	0.2728	ns

* statistically significant differences among the comparison groups.

ns: no statistically significant differences (Chi-square tests)

As shown in Table 1, there were significantly more surgical extractions in the Pre-iSTOP period (406/1188, 34.2%) than those in Post-iSTOP-1 (257/984, 26.1%) and Post-iSTOP-2 (269/1089, 24.7%) (p<0.05). The decrease in the amount of surgical extractions was associated with the establishment of a referral relationship with the oral surgery specialty clinic within our institution. There were no statistically significant differences in other treatment procedures among the three 3-momth periods.

The most common prescriptions for dental emergency patients were non-opioid and opioid analgesics, antibiotics, and antimicrobial mouth rinses (0.12% Chlorhexidine) (Table 1). A majority of the patients received pain medications in each of the 3-month periods: 1475/1921 (76.8%) in the Pre-iSTOP period, 1348/2011 (67.0%) in Post-iSTOP-1, and 1457/2272 (64.1%) in Post-iSTOP-2. For patients who were prescribed pain medications, 452/1475 (30.6%) received opioids analgesics in the Pre-iSTOP period, 190/1348 (14.1%) in Post-iSTOP-1, and 140/1457 (9.6%) in Post-iSTOP-2, signifying a statistically significant reduction in the number of opioid prescriptions after implementation of the mandatory PDMP (p<0.05). The frequency of non-opioid analgesic prescriptions did not change statistically significantly over the three study periods (p>0.05) (Table 1).

The odds for a patient who needed pain medication to receive opioid analgesics was reduced by 63% (OR 0.37, 95% CI: 0.31, 0.45) in Post-iSTOP-1, and by 76% (OR 0.24, 95% CI: 0.20, 0.30) in Post-iSTOP-2 as compared to the Pre-iSTOP period. Because less surgical extractions were performed in the two Post-iSTOP periods than in the Pre-iSTOP period, reduction in opioid drug prescription might be partly associated with decreased invasiveness of treatment procedures. The frequency of surgical extraction was therefore adjusted in the two Post-iSTOP periods based on that in the Pre-iSTOP period (34.2%) to account for the effect of treatment invasiveness. To accurately estimate the likelihood for a patient who had surgical extractions to receive a prescription of opioid analgesics, we further analyzed the prescription data of 404 patients who received surgical extractions in the Pre-iSTOP period, and found that 199 (or 49%) of them were prescribed opioid analgesics. We calculated the adjusted OR based on the fact that 49% of the patients who had surgical extractions received opioid analgesics. After adjustment for the frequency of surgical extractions, we found that the odds for a patient to receive opioid analgesics was reduced by 58% (OR = 0.42, 95% CI: 0.35, 0.51) in Post-iSTOP-1 and 72% (OR 0.28, 95% CI: 0.23, 0.34) in Post-iSTOP-2 as compared to the Pre-iSTOP period. The decreases in the odds of receiving opioid analgesics in the two Post-iSTOP periods remained to be statistically significant after adjusting for the frequency of surgical extractions (p<0.05).

Table 2 lists the frequency and quantity (numbers of pills) of opioid and non-opioid analgesics prescribed for dental emergency patients before and after implementation of the mandatory PDMP. Hydrocodone was the most frequently prescribed opioid analgesics, followed by codeine and oxycodone. Ibuprofen was the most frequently prescribed non-opioid analgesics, followed by acetaminophen. Following the implementation of the mandatory PDMP, there was an overall trend for decreasing opioid and increasing non-opioid analgesic prescriptions (Table 2). Total numbers of prescriptions for hydrocodone, codeine and oxycodone decreased significantly (p<0.05), while those for acetaminophen increased significantly (p<0.05). Prescriptions for ibuprofen increased by 19.8%, though it did not reach the level of statistical significance (p>0.05). By the end of the study, the total quantity of opioid analgesics prescribed in a 3-month period had reduced from 5096 to 1120 pills, signifying a 78% reduction in absolute quantity.

**Table 2:** Frequency and quantity (numbers of pills) of pain medication prescriptions for dental emergency patients before and after implementation of the mandatory PDMP.

		Pre-iSTOP		Post-iSTOP-1		Post-iSTOP-2	
		prescriptions	pills	prescriptions	Pills	prescriptions	pills
Opioids	Hydrocodone	378	4145	163	1393	106	827
	Codeine	51	650	24	222	30	251
	Oxycodone	23	301	3	24	4	42
	**Subtotal**	**452**	**5096**	**190**	**1639**	**140**	**1120**
Non-opioids	Ibuprofen	1000	22257	1051	23600	1198	25846
	Acetaminophen	23	467	107	1645	119	1992
	**Subtotal**	**1023**	**22724**	**1158**	**25245**	**1317**	**27838**
	**Total**	**1475**	**27820**	**1348**	**26884**	**1457**	**28959**

## Discussion

The findings of the present study indicate that the mandatory PDMP was effective in reducing prescriptions for opioid analgesics by dentists in a dental urgent care center. Following implementation of the mandatory PDMP, frequencies and quantities of prescriptions for opioid analgesics, represented by hydrocodone, oxycodone and codeine, decreased significantly, accompanied by a moderate increase in non-opioid analgesics represented by ibuprofen and acetaminophen. The frequency of opioid prescriptions was at least halved and the absolute quantity of opioid pills prescribed was reduced by 78%.

Dental pain is one of the most common types of bodily pain. Opioid analgesics are among the most frequently prescribed drugs by dentists [11–13]. Accordingly, dentists have contributed significantly to the prescription opioid epidemics by prescribing 12% of all immediate release opioid drugs in the United States, second only to family physicians in amounts of such prescriptions [14]. As immediate release opioids such as hydrocodone (e.g, Vicodin) and oxycodone (e.g. Percocet) are the most frequently abused prescription opioids [1, 14], dentists should always use caution when prescribing pain-relieving medications to minimize inadvertent dispensation of this type of opioids to drug-seekers in dental offices [12, 14–16]. As patients who abuse controlled substances often have poor oral health status [17, 18], they may be at higher risk of dental pain associated with pulpal and periodontal infections. They are therefore more likely to present themselves to emergency clinics and urgent dental care centers for treatment of dental pain. Abusers of prescription opioids may exaggerate their symptoms to deceive healthcare providers with the intention to seek and receive these drugs [3, 14, 19]. As dental pain is subjective and there is a lack of reliable objective measure for its severity, dentists have to rely on patient’s complaint to prescribe pain medications. In an urgent care setting, dentists often are not aware of a patient’s history of substance abuse unless the patient voluntarily reports it. Therefore, it is perceivable that some opioid analgesics are prescribed by dentists to patients who abuse them. The PDMP should be a very useful tool in helping dentists to identify patients who abuse prescription opioid drugs. The findings of the present study indicate a 78% reduction in quantity of opioid analgesics prescribed in a dental emergency clinic following implementation of the mandatory PDMP. However, such dramatic reduction in opioid prescriptions could not be fully explained by the numbers of dental urgent care patients who were suspected to abuse prescription opioid analgesics. “Chilling effect” of the PDMP, where practitioners refrain from prescribing controlled substances for fear of punitive actions against them from governing agencies, may result in decreased prescriptions of opioid analgesics for patients with chronic pain conditions [20, 21]. While such “chilling effect” may be a concern when practitioners need to prescribe a controlled substance in large quantities for an extended period of time for chronic pain, it is much less of a problem for a dentist who treat acute postoperative dental pain, a transient condition that usually requires only 1 to 3 days of pain management. We believe that a shift towards evidence-based practice is a more important reason for the decreased prescription of opioid analgesics. Current best research evidence indicates that Ibuprofen, either alone or in combination with acetaminophen, is more effective than commonly prescribed opioid analgesics in controlling postsurgical dental pain [22].

From empirical experiences, opioid analgesics such as those containing hydrocodone (such as Vicodin with hydrocodone 5mg plus acetaminophen 300mg, and Vicodin ES with hydrocodone 7.5mg plus acetaminophen 300mg) are often considered by dentists as “stronger” or more potent pain medications than non-opioid analgesics such as ibuprofen 400mg–600mg when prescribing for acute postoperative pain. Accordingly, hydrocodone plus acetaminophen has been found to be the most frequently prescribed analgesics following invasive dental procedures such as surgical extractions and third molar removals [11, 23], despite the lack of scientific evidence supporting opioid analgesics as the first choice medication for acute pain management. Numerous randomized clinical trials have established that non-steroidal anti-inflammatory drugs (NSAIDs) such as ibuprofen, either alone or in combination with acetaminophen, is superior to opioid analgesics such as oxycodone, either alone or in combination with acetaminophen [22, 24]. Assessed by the number needed to treat (NNT) to achieve adequate pain relieve in one patient, recent systematic reviews indicate that the NNT is 2.5 (95% CI 2.4–2.6) for ibuprofen 400mg alone, and 1.6 (1.5–1.8) for ibuprofen 200mg plus acetaminophen 500mg, but 4.6 (2.9–11.0) for oxycodone 15mg alone, and 2.7 (2.4–3.1) for oxycodone 10mg plus acetaminophen 650mg [25–27]. There is no study that directly compared hydrocodone plus acetaminophen to ibuprofen for acute postoperative pain control. However, it has been shown that the NSAID ketorolac 10mg alone was superior to hydrocodone 10mg plus acetaminophen 1000mg [28] for acute postoperative pain. As the effectiveness of ibuprofen 400mg (NNT = 2.5, 95%CI 2.4–2.6) is at least comparable to that of ketorolac 10mg (NNT = 2.6, 95% CI = 2.3–3.1) [29], it could be concluded that ibuprofen 400mg is more effective than the Vicodin and Vicodin ES formulations (hydrocodone 5mg—7.5mg plus acetaminophen 300mg) for acute postoperative pain. Acetaminophen 500mg (NNT 3.5) or 1000mg (NNT 3.6) alone or acetaminophen 300mg plus codeine 30mg (NNT = 6.9) was less effective than ibuprofen 200mg (NNT 2.7) or 400mg (NNT 2.5), or ibuprofen 200mg plus acetaminophen 500mg (NNT 1.6) [25, 26, 30, 31]. These evidences have led to the recommendations of using ibuprofen 400mg to 600mg alone for mild to moderate pain and ibuprofen 400mg to 600mg plus acetaminophen 500mg for moderate to severe pain after invasive dental surgery in clinical practices [22].

Hydrocodone in combination with acetaminophen was by far the most frequently prescribed opioid analgesics for dental emergency patients in this study, which is consistent with several other reports that showed that dentists in the United States overwhelming favored this drug for acute postoperative pain [11, 13, 23]. The reason for such favoritism is unclear, as there is no evidence to show that hydrocodone plus acetaminophen is superior to other opioid or non-opioid analgesics. Hydrocodone plus acetaminophen was found to be inferior not only to NSAIDs but also to oxycodone plus ibuprofen or acetaminophen for acute postoperative pain [28, 32]. Following implementation of the mandatory PDMP, the numbers of prescriptions for hydrocodone and other opioid analgesics did decrease significantly while those for non-opioid analgesics increased (Table 2). This change in prescription pattern conforms to the evidence-based prescription recommendations for acute postoperative pain [22, 24]. It is likely that the mandatory PDMP has prompted dentists to reassess their prescription practices for acute postoperative pain and select more appropriate analgesics for their patients based on the best evidence available.

The primary purpose of the mandatory PDMP is to reduce overprescribing controlled substances by health practitioners and to curtail the diversion and abuse of opioid analgesics. However, it should not restrict the access to opioid analgesics for patients who truly need them. The findings of the present study show that this program did cause a significant reduction in prescription of opioid analgesics for dental emergency patients. As dental pain may significantly impact the quality of life of our patients, it is important that they receive adequate pain control following an invasive dental procedure. One of our concerns is that the dramatic reduction in opioid analgesics after implementation of the mandatory PDMP may jeopardize pain treatments for dental urgent care patients. All patients treated at the urgent care center received verbal and written postoperative instructions, and we expected that the patients would have returned to our clinic for further treatment if they did not receive adequate pain control after the initial visit. The number and frequencies of return visits, represented by the ADA codes D0113, D0115, D0117 and D9430, would have increased following implementation of the mandatory PDMP if curtailing prescriptions for opioid analgesics had jeopardized pain treatments for these patients. Our data show that the frequencies of return visits were 5.8%, 4.3%, and 4.8% for the three study periods, respectively, which suggests that lack of opioid analgesics did not mean poor pain control for the dental emergency patients. Nevertheless, it is important to provide training for dentists on best practices for acute pain treatments and minimize the risk of underprescribing pain medications in light of the mandatory PDMP [33]. A prospective study is warranted to investigate the definitive impact of changing prescription pattern on the management of acute postoperative pain in dental practices.

## Conclusion

Frequencies and quantities of prescriptions for opioid analgesics, such as hydrocodone, oxycodone and codeine, decreased significantly, while those for non-opioid analgesics, such as Ibuprofen and acetaminophen, increased moderately following implementation of the mandatory PDMP. As current research evidence indicates that non-opioid analgesics (ibuprofen and acetaminophen) are more effective than opioid analgesics (hydrocodone, oxycodone and codeine) in controlling acute dental pain, such change in prescription pattern may represent a shift towards the evidence-based prescription practices for patients with acute dental pain.
